# Extrapulmonary tuberculosis in non-human immunodeficiency virus-infected adults in an endemic region

**DOI:** 10.4103/1817-1737.33700

**Published:** 2007

**Authors:** Mustafa Kürşat Özvaran, Reha Baran, Meltem Tor, Ilknur Dilek, Dilay Demiryontar, Sibel Arinç, Nil Toker, Efsun Uğur Chousein, Özlem Soğukpinar

**Affiliations:** *Department of Pulmonology, SSK Sureyyapasa Center for Chest Disease and Thoracic Surgery, Istanbul, Turkey*; * *Department of Pulmonology, Kara Elmas University, Medical School Zonguldak, Turkey*

**Keywords:** Extrapulmonary tuberculosis, gender, incidence, tuberculosis

## Abstract

**AIMS:**

Extrapulmonary tuberculosis (EPTB) still constitutes an important clinical problem. We aimed to evaluate the incidence and features of extrapulmonary tuberculosis.

**MATERIALS AND METHODS:**

We retrospectively evaluated 14,266 tuberculosis patients diagnosed between January 1999 and December 2003 in a tertiary care hospital in Istanbul. As many as 2,435 patients (17.1%) with EPTB were evaluated for the incidence and features.

**RESULTS:**

Of the 14,266 patients, 4,154 were female (29%) and 10,112 were male (71%) and were aged between 14 and 86 years with a mean age of 35 ± 14 years. As many as 660 (17.9%) patients were diagnosed as EPTB in 1999, 568 (17.8%) in 2000, 357 (13.7%) in 2001, 462 (22%) in 2002 and 388 (14.5%) in 2003. EPTB presented most commonly as pleurisy (66%), followed by lymphadenitis (23%). Lymphadenitis and pleurisy were more commonly observed among female TB patients (60%) and among male TB patients (59%) respectively. EPTB showed a significant female predilection (26.8%) compared to male patients (13.1%). Multi-organ involvement was observed in 37 (1.5%) patients (two organs in 33 and three organs in 4). As many as 197 (8%) EPTB cases had pulmonary tuberculosis simultaneously.

**CONCLUSIONS:**

EPTB still constitutes an important clinical problem. The rates of EPTB have remained constant despite the decline in pulmonary tuberculosis cases. In the current study, we present our experience of the incidence and features of EPTB patients without HIV infection. In this study, EPTB cases constituted one-fifth of all tuberculosis cases presented to our center in the study period.

Tuberculosis (TB) is a serious health problem worldwide and constitutes the main infectious cause of death.[1] It can involve any organ system in the body. Although pulmonary tuberculosis is the most common presentation, extrapulmonary tuberculosis (EPTB) still constitutes an important clinical problem. EPTB, by definition, is the isolated occurrence of TB at body sites other than the lung. Extrapulmonary organ involvement of TB is estimated as 10-34% of patients who are not infected with human immunodeficiency virus (HIV), whereas the frequency is about 50-70% in patients infected with HIV.[2–3] The rates of EPTB have remained constant despite the decline in pulmonary tuberculosis cases. The most common sites of extrapulmonary involvement were reported as pleural and lymph node.[4–5]

In the current study, we present our experience of the incidence and features of EPTB patients without HIV infection.

## Materials and Methods

Of the 14,266 TB patients, 2,435 (17.1%) patients with EPTB were included in the study. Patients presented between January 1999 and December 2003 at SSK Sureyyapasa Center for Chest Disease and Thoracic Surgery, a tertiary care hospital in Istanbul, Turkey, were reviewed retrospectively. Only patients with an extrapulmonary site of infection were included in the study. Miliary TB cases and sole pulmonary TB cases and multidrug-resistant patients' cases were excluded. EPTB and pulmonary TB were defined as follows: (1) Identification of *Mycobacterium tuberculosis* through Ziehl-Neelsen acid fast stain and culture in Lowenstein-Jensen or BACTEC media in a tissue or specimen from a site other than lung parenchyma, in association with clinical and/or imaging findings compatible with infection locally, (2) EPTB and pulmonary TB with negative cultures were defined as clinical, laboratory, imaging and/or histopathological evidence of mycobacterial infection in a site other than hilar lymph nodes or lung parenchyma, in association with a positive tuberculin skin test reaction and/or history of exposure to tuberculosis and exclusion of other diseases and response to empirical antituberculosis treatment. We evaluated the clinical presentation of EPTB cases, including demographic features, organ involvement and coexisting pulmonary TB.

All patients had HIV test in our hospital by macro-eliza method (Beckman Coulter, Inc., CA, USA). None of the patients had HIV co-infection. Descriptive statistics (mean, standard deviation, percentage) and Chi-square test were used.

## Results

We identified 2,435 EPTB cases (17.1%) from the 14,266 TB patients who presented to our center during the study period. TB cases included 10,112 (71%) male and 4,154 (29%) female patients aged between 14 and 86 years with a mean of 35 ± 14 years. Table 1 shows age and gender distribution according to years. Table 2 shows EPTB and organ involvement according to years. EPTB with pulmonary TB was observed in 197 patients (8%). Thirty-seven patients (1.5%) with EPTB had multi-organ involvement (two organs in 33 and three organs in 4).

Table 1The distribution of age and gender in tuberculosis by time

**Table:** 

Tuberculosis	1999		2000		2001		2002		2003		Total	
						
	n	%	n	%	n	%	n	%	n	%	n	%
Female	971	6.8	916	6.4	699	4.9	672	4.7	896	6.3	4154	29.1
Male	2717	19.1	2276	16	1903	13.3	1429	10	1787	12.5	10112	70.9
Total	3688	25.9	3192	22.4	2602	18.2	2101	14.7	2683	18.8	14226	100

The distribution of age and gender in extrapulmonary tuberculosis by time

**Table:** 

EPTB	1999		2000		2001		2002		2003		Total	
Age	14-79 mean 33		15-79 mean 35		15-77 mean 36		15-79 mean 35		15-86 mean 36		14-86 mean 35	
						
	n	%	n	%	n	%	n	%	n	%	n	%
Male	407	11	334	10.5	160	6.1	244	11.6	177	6.6	1322	9.3
Female	253	6.9	234	7.3	197	7.6	218	10.4	211	7.9	1113	7.8
Total	660	17.9	568	17.8	357	13.7	462	22	388	14.5	2435	17.1

EPTB - Extrapulmonary tuberculosis

The most common site of extrapulmonary involvement was the pleura, followed by the lymph nodes. Of the 1,637 (67%) patients with pleural disease, 664 (41%) were female. Of the 567 (23%) patients with lymphadenitis, 342 (60%) were females. Lymph node involvement occurred with significantly higher frequency in women (*P* < 0.001), while pleural disease was significantly higher in men (*P* < 0.001). EPTB showed a significant female predilection (26.8% *vs.* 13.1%) (*P* < 0.001).

## Discussion

TB is again on the rise all over the world with increasing incidence of multidrug-resistant TB and HIV disease. It is also an increasing health problem because of the immigrants from underdeveloped countries, where it is more common. Extrapulmonary tuberculosis cases have been largely due to HIV co-infection. The rates of EPTB have remained constant despite the decline in pulmonary tuberculosis cases. EPTB accounts for up to one-third of all cases.[6] In the present study, we aimed to investigate the ratio of EPTB in our endemic region where HIV infection is uncommon. We found 17.1% of TB patients presented with EPTB, among all TB cases. EPTB was more common in females than in males. The most common sites of extrapulmonary involvement were the pleura, followed by the lymph nodes.

*Mycobacterium tuberculosis* is presumed to spread in sequence from primary lung site to various organs and tissues of the body. Therefore, tuberculosis can cause disease in all organs and mimic various diseases of these organs. There have not been enough studies about the incidence of EPTB in Turkey; some studies in Holland and England reported 32 and 34% of all cases respectively.[7–8]

A successful TB control program and treatment caused a decrease in pulmonary TB despite an increase in ratio of EPTB being reported as 21% in Western Europe, 10% in Eastern Europe.[9] It was shown that in USA EPTB ratio was 17.5% in 1986, while it was only 8% in 1964.[4] In our study, we recognized no decrease in ratios in the fifth year, as also confirmed by other studies.

Many studies predicted that mostly pleura and regional lymph nodes are likely to be involved. Gonzalez *et al.* reported[4] 538 EPTB cases (28.6%) in a total of 1,878 enrollees. The most common sites of infection were lymph nodes (43%) and pleura (23%). In the same study, African American ethnicity was found to be an independent risk factor for EPTB. Recently Ilgazli *et al.* reported 636 cases with EPTB, out of which 56.3% were lymph node tuberculosis, followed by pleural tuberculosis (31.1%).[10]

A study from England showed that lymph node involvement was the most common site in the body.[7] However, a study from Hong Kong showed the most common sites were pleura, followed by lymph node; and one-third of all cases had pulmonary tuberculosis. In our series, 8% of cases had pulmonary tuberculosis simultaneously. A study in Holland showed that the most common sites of EPTB were pleura and lymph node, equally (17%).[8] Maltezou and his colleagues found the lymph node was the most common site of EPTB in children and EPTB constituted 9% of all cases with TB.[6] Lymph node involvement in EPTB was correlated with HIV co-infection, female gender, young age and Asian race.[11] Our study showed lymph node involvement was the second most common site. In this study, none of the patients were HIV infected and of childhood age, but lymph node involvement was more frequently observed in female patients than in male.

**Table 2:** Involved organ in patients with extrapulmonary tuberculosis according to years

	1999		2000		2001		2002		2003		Total	
						
Site	n=669	%	n=580	%	n=367	%	n=468	%	n=392	%	n=2476	%
Pleura	457	68	397	69	229	62	302	65	252	64	1637	67
Lymph node	143	21	130	22	91	25	97	21	106	27	567	23
Liver	1	0.2	1	0.2	0	0	0	0	0	0	2	0.0008
Bowel	1	0.2	1	0.2	1	0.3	1	0.2	2	0.5	6	0.2
Small bowel	0	0	1	0.2	0	0	0	0	0	0	1	0.0004
Anal-rectum	0	0	0	0	0	0	1	0.2	0	0	1	0.0004
Peritoneum	19	3	12	2	13	4	13	3	13	3	70	3
Parotid	0	0	0	0	2	0.6	0	0	1	0.3	3	0.1
Tongue	0	0	0	0	1	0.3	0	0	0	0	1	0.0004
Nasal pharynx	1	0.2	0	0	0	0	0	0	0	0	1	0.0004
Thyroid	0	0	0	0	0	0	1	0.2	0	0	1	0.0004
Adrenal	0	0	0	0	0	0	1	0.2	0	0	1	0.0004
Endometrium	4	0.6	0	0	0	0	0	0	0	0	4	0.2
Ovary	1	0.2	1	0.2	2	0.6	0	0	0	0	4	0.2
tubes	1	0.2	2	0.4	1	0.3	1	0.2	0	0	5	0.2
testis	0	0	1	0.2	0	0	2	0.4	0	0	3	0.1
Urinary tract	2	0.3	2	0.4	4	1.1	2	0.4	2	0.5	12	0.5
Prostate	0	0	0	0	1	0.3	1	0.2	0	0	2	0.0008
Breast	0	0	3	0.5	0	0	4	0.9	0	0	7	0.3
Larynx	9	1	5	0.9	1	0.3	4	0.9	2	0.5	21	0.9
Eye	0	0	1	0.2	0	0	0	0	0	0	1	0.0004
Pericardium	3	0.5	4	0.7	2	0.6	8	1.7	3	0.8	20	0.8
Skin	0	0	3	0.5	1	0.3	6	1.3	1	0.3	11	0.4
Brain(tbrklm)	3	0.5	0	0	2	0.6	0	0	0	0	5	0.2
Meninges	2	0.3	2	0.4	1	0.3	3	0.6	0	0	8	0.3
Spleen	1	0.2	2	0.4	0	0	0	0	0	0	3	0.1
Joint	0	0	0	0	0	0	1	0.2	0	0	1	0.0004
Bone	2	0.3	4	0.7	4	1.1	5	1.1	0	0	15	0.6
Vertebrae	11	2	6	1	8	2.2	11	2.4	6	1.5	42	2
Soft tissue	4	0.6	2	0.4	3	0.8	6	1.3	0	0	15	0.6
Bone marrow	4	0.6	0	0	0	0	1	0.2	0	0	5	0.2

In our study, EPTB was more common in females than in males (26.8% *vs.* 13.1%). In our cases, male-to-female ratio was 71% and 29% respectively. This ratio is consistent with the literature. Gonzalez *et al.* indicated that EPTB had been more commonly observed in females (218 female EPTB / 396 female TB cases) as compared to males (320 EPTB cases / 944 male TB cases) in Houston.[4] Noertjojo *et al.*[5] showed that TB was more common in males than in females but EPTB has a predilection for females at all ages. A study in Turkey showed EPTB was more common in females than in males.[12] Abdomen is also one of the most common sites of EPTB involvement. Before the antituberculosis drug era, the ratio was 55-90%, which later declined to 25%.[11] Peritonitis is the most common presentation of abdominal involvement, followed by ileocecal involvement.[13] Bayramicli *et al.* showed that the most common sites in abdomen were small bowel (48%), followed by peritoneum (35%) and lymph nodes (17%).[11] In our series, peritonitis (3%) was found as the most common site in abdomen.

TB of the genitourinary tract was reported as the second most common disease form of extrapulmonary TB.[14] The most common site of bone TB was the vertebral column, and the involvement is generally monoarticular. A study in the US found, again, pleura is the most common site (25.8%), followed by lymph node (17.4%), bone (14.3%) and genitourinary (14.6%) in between 1982 and 1986.[15] In our study, we did not find similar distribution for genitourinary TB. This can be explained by a bias in terms of organ involvement, as our center is a tertiary referral chest hospital and not all EPTB cases are referred to our center. TB of bone was the fourth most common site in EPTB in our series.

In this study, we found that 1.5% of EPTB patients had multi-organ involvement. During the primary infection of TB, bacteria can spread to all organs in the body. Therefore, EPTB can be observed in different organs at the same time.

In this study, we found one in every five TB patients had extrapulmonary TB. We also found the EPTB was two times more common in females than in males. Clinical signs; laboratory, radiological and endoscopic methods; or bacteriological and histopathological findings help in the diagnosis of EPTB, although laboratory methods might at times be challenging for EPTB compared to pulmonary TB. So clinical suspicion based on chest radiography, localized or systemic symptoms and several laboratory parameters and rapid diagnostic methods expedite the treatment of such cases.
